# Investigating the Potential of a Conversational Agent (Phyllis) to Support Adolescent Health and Overcome Barriers to Physical Activity: Co-Design Study

**DOI:** 10.2196/51571

**Published:** 2024-01-31

**Authors:** Richard Moore, Abdel-Karim Al-Tamimi, Elizabeth Freeman

**Affiliations:** 1 Sheffield Hallam University Sport and Physical Activity Research Centre / Advanced Wellbeing Research Centre Sheffield United Kingdom; 2 Department of Computing Sheffield Hallam University Sheffield United Kingdom; 3 Department of Psychology, Sociology & Politics Sheffield Hallam University Sheffield United Kingdom

**Keywords:** physical activity, inactivity, conversational agent, CA, adolescent, public health, digital health interventions, mobile phone

## Abstract

**Background:**

Conversational agents (CAs) are a promising solution to support people in improving physical activity (PA) behaviors. However, there is a lack of CAs targeted at adolescents that aim to provide support to overcome barriers to PA. This study reports the results of the co-design, development, and evaluation of a prototype CA called “Phyllis” to support adolescents in overcoming barriers to PA with the aim of improving PA behaviors. The study presents one of the first theory-driven CAs that use existing research, a theoretical framework, and a behavior change model.

**Objective:**

The aim of the study is to use a mixed methods approach to investigate the potential of a CA to support adolescents in overcoming barriers to PA and enhance their confidence and motivation to engage in PA.

**Methods:**

The methodology involved co-designing with 8 adolescents to create a relational and persuasive CA with a suitable persona and dialogue. The CA was evaluated to determine its acceptability, usability, and effectiveness, with 46 adolescents participating in the study via a web-based survey.

**Results:**

The co-design participants were students aged 11 to 13 years, with a sex distribution of 56% (5/9) female and 44% (4/9) male, representing diverse ethnic backgrounds. Participants reported 37 specific barriers to PA, and the most common barriers included a “lack of confidence,” “fear of failure,” and a “lack of motivation.” The CA’s persona, named “Phyllis,” was co-designed with input from the students, reflecting their preferences for a friendly, understanding, and intelligent personality. Users engaged in 61 conversations with Phyllis and reported a positive user experience, and 73% of them expressed a definite intention to use the fully functional CA in the future, with a net promoter score indicating a high likelihood of recommendation. Phyllis also performed well, being able to recognize a range of different barriers to PA. The CA’s persuasive capacity was evaluated in modules focusing on confidence and motivation, with a significant increase in students’ agreement in feeling confident and motivated to engage in PA after interacting with Phyllis. Adolescents also expect to have a personalized experience and be able to personalize all aspects of the CA.

**Conclusions:**

The results showed high acceptability and a positive user experience, indicating the CA’s potential. Promising outcomes were observed, with increasing confidence and motivation for PA. Further research and development are needed to create further interventions to address other barriers to PA and assess long-term behavior change. Addressing concerns regarding bias and privacy is crucial for achieving acceptability in the future. The CA’s potential extends to health care systems and multimodal support, providing valuable insights for designing digital health interventions including tackling global inactivity issues among adolescents.

## Introduction

### Background

There is indisputable evidence supporting the positive effects of regular physical activity (PA) on the physical and emotional well-being of adolescents [1], enhanced cognitive function [2], and improved educational attainment [3,4]. The UK government recommends that children and young people engage in 60 minutes of PA per day, with 30 minutes taking place at school and an additional 30 minutes outside of school hours. However, despite this guideline, only 57% of children participate in ≥30 minutes of PA outside of school, compared with 40% during school hours [5].

Inactivity is a global challenge, with a considerable number of adolescents worldwide failing to engage in sufficient PA [6]. It is crucial that adolescents maintain a consistent level of PA during adolescence as this may lead to healthy habits that extend into adulthood, lowering the risk of inactivity in later years and reducing the possibility of developing hypokinetic conditions [7-9]. There is a consensus that evidence-based approaches involving multicomponent interventions targeted at inactive adolescents are needed on a global scale [10-12].

Although schools provide equal opportunities for adolescents to participate in PA, making them valuable settings to focus resources [12], multiple barriers exist that prevent schools from allocating adequate time and resources to PA programs [13,14]. In addition, various barriers relating to physical, psychological, social, and environmental factors hinder PA participation in the adolescent population [15-17]. It is generally accepted that limited progress has been made in supporting adolescents to overcome these barriers, necessitating the development of innovative interventions [12]. This rationale underlies the launch of the Global Action Plan on PA in 2018 by the World Health Organization, aimed at promoting and supporting adolescents in achieving the recommended levels of PA [18]. As part of this plan, digital approaches were advocated, emphasizing that interventions should prioritize understanding and addressing barriers that prevent adolescents’ engagement in PA. By doing so, interventions can be effectively designed to facilitate sustainable changes in the PA habits and behaviors of adolescents [15-17].

Digital health interventions have emerged as a promising approach to promoting PA and overall well-being through digitally delivered support, providing PA education and motivational guidance [19]. However, the effectiveness of these interventions in promoting PA has yielded mixed results [20,21]. A systematic review focusing on digital interventions for PA among young people revealed positive changes in PA levels and attitudes toward PA [22]. These positive changes were attributed to numerous factors such as web-based education, goal setting, self-monitoring, parental involvement, and gamification and personalization [22]. In particular, personalization has emerged as a significant feature in engaging adolescents through digital health interventions [23]. Tailoring interventions to individual needs and preferences is crucial for promoting PA and overcoming barriers to participation. There is also some evidence of the positive engagement of young people in formal (eg, school) and informal (eg, home) settings to develop knowledge, skills, and behaviors related to PA [22]. This shows that locations, such as school and home, hold potential as promising venues for implementing such interventions.

Conversational agents (CAs) have emerged as promising tools for promoting PA, especially among adolescents, using popular digital platforms such as social media, web interfaces, and mobile apps [24-26]. These agents are accessible at any time of the day, are nonjudgmental, and can be accessed on an array of digital devices. CAs are the preferred choice for individuals seeking immediate web-based support and for those who are hesitant about seeking in-person assistance.

Systematic reviews have examined CA effectiveness in promoting PA, healthy eating, and weight loss [27,28]. Nevertheless, despite encouraging results, the efficacy of these interventions remains inconclusive owing to constraints in outcome measurement and reporting [28]. There is also a dearth of evidence regarding how such CAs have been used to help adolescents overcome barriers to PA, and there is a need for new conversational artificial intelligence (AI) approaches that draw insights from existing theories to ensure they are evidence based and effective [17,29].

To optimize CAs and maximize user engagement, previous studies have emphasized the importance of effective persona design and the integration of principles from user-centered design [30] and human-computer interaction [28,31]. However, previous research focusing on usability and feasibility has reported moderate results, highlighting challenges such as repetitive content, high attrition rates, technical difficulties, and concerns regarding safety and privacy [27]. Although rule-based approaches are commonly used, more successful outcomes have been achieved with unconstrained methods that allow for natural language input and personalized interaction. These methods offer opportunities for establishing relational and persuasive capacities [28] with adolescents, which are particularly important when delivering person-centered behavior change interventions.

Recent advancements in large language models (LLMs) as well as the advent of generative AI show promise in enhancing CAs by improving user engagement and satisfaction, potentially surpassing human performance [32]. Their ability to understand and generate natural language with complexity and accuracy allows for sophisticated conversations, including emotion detection, contextual understanding, and personalized responses [33,34]. LLMs offer automatic content generation, increasing scalability and cost-effectiveness [33,34]. All of these features could potentially enhance CA quality and improve adherence in the future; however, concerns about bias, misinformation, privacy, and security need to be addressed before using them safely with adolescents [33,34]. In any health-related context, it is of paramount importance that the information delivered is not only accurate and trustworthy but also firmly rooted in evidence-based practices, curated in collaboration with domain experts. Consequently, as consideration focuses on the future applications of LLMs and generative AI in this sphere, it becomes imperative to place our faith in models that are not only trustworthy but also transparent, providing a clear and comprehensive audit trail for their decision-making processes. This ensures that the highest standards of quality and reliability are maintained, thereby safeguarding the integrity of health-related information and services.

### Summary

This study introduces the co-design and evaluation of a pilot CA called “Phyllis” to evaluate its proof of concept. Its objective is to assist adolescents in overcoming 2 barriers to PA (ie, confidence and motivation) and offer an alternative digital solution to support students and promote PA. Building upon the findings of a previous study, which identified 52 barriers to adolescents’ PA participation, appropriate intervention functions and behavior change tools were identified to provide support for each barrier [17]. A theoretical framework designed for this study and the existing behavior change model also inform the CA [29] and both aim to aid the development of CAs in promoting PA.

The hypothesis for the study is that a CA can support adolescents to overcome barriers to PA and be perceived by adolescents as being a tool to help them increase their confidence and motivation to participate in PA. The objectives of the study are as follows:

- Co-design a CA in collaboration with adolescents, incorporating the model by Zhang et al [29] and a theoretical framework designed for this study to assess its proof of concept.
- Demonstrate the CA’s ability to understand user input related to one of the 52 barriers to PA identified in the previous study [17].
- Evaluate the usability and acceptability of the CA among adolescents.
- Assess the perceived effectiveness of the solutions to barriers provided by the CA.

## Methods

### Overview

In this section, we present a comprehensive and systematic methodology used for the co-design and evaluation of the CA. The methodology includes 3 key phases: phase 1 focuses on co-designing the CA with adolescents to ensure the agent’s relevance and user-centeredness. In phase 2, the development of the CA and its dialogue is presented, incorporating persuasive and relational capacity elements. To facilitate intelligent and contextually relevant interactions, a natural language understanding (NLU) model and knowledge model are integrated into the cognitive system. Finally, in phase 3, we detail the mixed methods evaluation used to comprehensively assess the CA’s effectiveness and user experience with adolescents.

### Co-Design of the CA

#### Phase 1: Understanding User Background and Designing CA Characteristics and Persona

To participate in the co-design process, all students aged 11 to 13 years were required to apply through their school and meet the following selection criteria:

- Students perceived that they did not currently meet the UK government recommendations for participating in PA for 60 minutes per day over the course of a week.
- They were interested in participating in PA more often.
- In their application, they stated barriers to participation that had prevented them from being more physically active.

All interested students were given an information sheet and provided written consent to participate. The following is an example of an application from a student who participated in the co-design, highlighting the importance that students felt in participating in the research:

Sir, I think I would be great for this role because I have always wanted to do sports and be more fit, but I have always stopped myself because I thought I wouldn’t be good enough, I would embarrass myself, just fail altogether or I would be too scared and think that it was too hard. I think that this opportunity would bring out the best in me and give me another chance to build a better me. I also stop myself from the joy of joining in because I have always thought people would judge me and I would always get scared that I wouldn’t be perfect. Now you might be thinking why should choose this girl she has only listed things that she is bad at and why but one thing that Is that I love improving myself and with this once in a lifetime opportunity I could make a fresh start.

The primary objective of phase 1 of the study was to gain insights into the user background of the co-design participants, including sociodemographic characteristics (eg, age and sex), behavior determinants (eg, attitude toward PA), and behavior habits (eg, PA and CA use) [29].

To accomplish this, a 2-hour workshop was conducted at Wickersley School and Sports College, located in Rotherham, South Yorkshire, where the students were based. During the workshop, the following co-design activities were conducted with the students, following the principles of user-centered design [30,35]:

- Icebreaker activity: this involved a demonstration of a robot and an introduction by the research team.
- A 30-minute focus group discussion with the students, audio recorded, thematically analyzed [36], and covering the following topics:
  Participation in PA, barriers faced, and overall experiences related to PA
  Exploration of how students receive support regarding PA, including the use of resources (eg, digital platforms) to obtain information or support
  Expectations and past experiences with CAs, assessing their acceptability
  A brief overview of the potential road map for the CA’s design and an explanation of how students would contribute to the process

The second part of phase 1 involved the completion of a co-design workbook by the participating students. This workbook was specifically designed for the project and served to gather both quantitative and qualitative data on user background and design preferences [29]. It aimed to capture information regarding various aspects of the CA’s design, including the dialogue system infrastructure (eg, buttons or open text input), media content delivered through conversation (eg, videos, documents, and text), and anthropomorphic cues (eg, identity, name, and sex) of the CA. In addition, it provided content related to building relational capacity specific to this type of CA, such as social dialogue (eg, greetings and small talk) and self-disclosure (eg, discussing the CA’s development). The following presents a summary of the data collected during this stage of the co-design process:

- Students’ age, sex, and levels of PA
- Quantifying the barriers to PA that students faced
- Detailing their emotions toward PA using the wheel of emotions by Plutchik [37]
- Using Leary’s [38] Interpersonal Complex to identify the CAs personality along 2 dimensions: dominance (horizontal) and agreeableness (vertical)
- Identifying content that students would prefer to access via the CA (ie, conversational, videos, weblinks to other content, documents, and images)
- Identifying the preferred type of persona and key characteristics that the CA should possess

#### Phase 2: Developing Relational and Persuasive Conversational Capacity

##### Persuasive Capacity

To enhance the CA’s understanding of adolescents’ habits and behaviors and facilitate effective intervention design, it is crucial for all PA interventions to be grounded in existing evidence and theory. This phase of the study draws upon a theoretical framework that details a theory-based approach to designing CAs to support adolescents in overcoming barriers to PA. The framework used in this study is underpinned by the capability, opportunity, motivation, and behavior (COM-B) model and the Theoretical Domains Framework (TDF). Identified barriers were coded using the COM-B [39] model and TDF [40] to comprehend both the origins of adolescent behaviors and the factors influencing them. Furthermore, this approach ensured the appropriate selection of evidence-based intervention functions, policy factors, and behavior change tools for each behavior using the Behavior Change Technique Taxonomy [41]. This process involved choosing behavior change tools that could be effectively delivered conversationally through a CA [17]. The theoretical framework played a pivotal role in informing the cognitive system, including the knowledge model incorporating the study findings [17] and the conversational engine (ie, the NLU model) to accurately understand and respond to barriers input by adolescents.

The next step in the process was to evaluate the feasibility and usability of the approach and evaluate the changes resulting from the use of the CA. For this purpose, 2 CA modules were developed by the research team, specifically targeting the 2 primary barriers identified by the students: a lack of “confidence” and “motivation” to be physically active. The confidence module was designed based on the theory of planned behavior [42,43] and incorporated 3 evidence-based behavior change tools identified from a previous study [17], namely problem-solving, verbal persuasion about capability, and self-talk. These tools were developed based on 4 guiding principles:

- Setting goals: it is important to always have a goal and push oneself out of the comfort zone.
- Diverse goals: building confidence involves setting different types of goals to enhance overall confidence.
- Broad perspective: individuals with higher levels of self-efficacy can look at the bigger picture and go beyond short-term setbacks.
- Reframing setbacks: developing self-efficacy is a gradual process that may take years to reframe one’s mindset.
- Failure is inevitable, but it should be viewed as unimportant. What matters is how one chooses to deal with failure and the lessons learned from it overall.

During the conversation, adolescents were prompted to answer the following questions. They were then given the opportunity to review their answers on the screen and receive a copy of their responses via email. This provided students with the opportunity to revisit and follow up on the actions they had stated.

- How confident are you when it comes to PA. Can you rate your answer from 1-10 with 1 being “very low,” 5 being “okay,” and 10 being “very confident.”
- When you feel confident where are you?
- Do you feel more confident being active with others or by yourself?
- And what motivates you more–being active with others or by yourself?
- When you feel most comfortable–what activity are you doing?
- If you think that activity is exercise related (eg, skipping) how could you do it more within your week? If that activity is not exercise related (eg, drawing) how could you add an element of exercise to it? (eg, standing whilst drawing, jumping on the spot every 10 min whilst drawing, etc).
- Based on what you know now, how can you bring activity into your weekly routine more?

The motivation module was developed using 2 behavior change tools: instructions on how to perform the desired behavior and behavior rehearsal or practice. Students were given a series of information on how to be more physically active and how to build small sessions (ie, 15 min) of PA into their day. These activities could be undertaken by any able-bodied person at no cost. Further information was given about how activity could be achieved despite educational pressure and time constraints and how PA can improve attainment. In addition, links to Sport England websites were provided to give young people further information on how to increase their PA levels in various environments, such as at home or outdoors. Useful information was provided on why PA is important and how often we should aim to engage in it.

##### Relational Capacity

To foster relational capacity, it is essential to design conversations that are evidence based, are expert led, and incorporate user feedback. However, there is a lack of empirical evidence regarding effective conversation design for CAs. To ensure that the CA is engaging and effective while adhering to the principles of conversation design, module design followed the happy and detailed conversation design process provided by the Conversation Design Institute [44]. This involved the inclusion of appropriate greetings, small talk, and module messaging that incorporated the elements of social dialogue, empathy, and humor. Sample dialogues were evaluated, and iterative refinements were performed using this process. Theoretical principles [17,29], along with the concepts of user-centered design [30] and human-computer interaction [31], were also considered.

To enable effective natural language interaction between humans and the CA, the researchers developed an NLU model [45]. They assigned at least one intent to each identified barrier from a previous study [17] to train the NLU model. For each intent, a minimum of 15 utterances were manually generated by the researchers and uploaded to Google Dialogflow’s NLU engine to train the data [46]. The training process involved providing the system with a diverse range of conversational data, allowing it to learn and comprehend various patterns, intents, and entities relevant to the specific domain. Through iterative training and refinement, the Dialogflow NLU model was optimized to accurately understand and interpret user input, forming a solid foundation for the subsequent phases of CA development and evaluation [47].

The NLU model represents a hybrid approach, falling between the spectrum of fully open-ended models, such as ChatGPT, and strictly closed branching–dialogue systems. Instead of strictly adhering to predetermined dialogue paths or being entirely unrestricted, the NLU model uses a nuanced methodology. It focuses on the interactions between the CA and students through several natural language processing techniques. Specifically, the NLU model is adept at comprehending and interpreting user inputs in natural language. It identifies the intent behind user queries and recognizes key entities mentioned in the text. Unlike rigid closed systems, the model does not follow a fixed script. Instead, it uses this understanding of user intent and entities to dynamically route the conversation. Although predefined dialogues are in place to guide the conversation, these paths are adaptable based on the context of the conversation and the user’s input. This flexibility ensures a more engaging and personalized interaction while still maintaining a degree of structure within the conversation flow.

**Figure 1:**
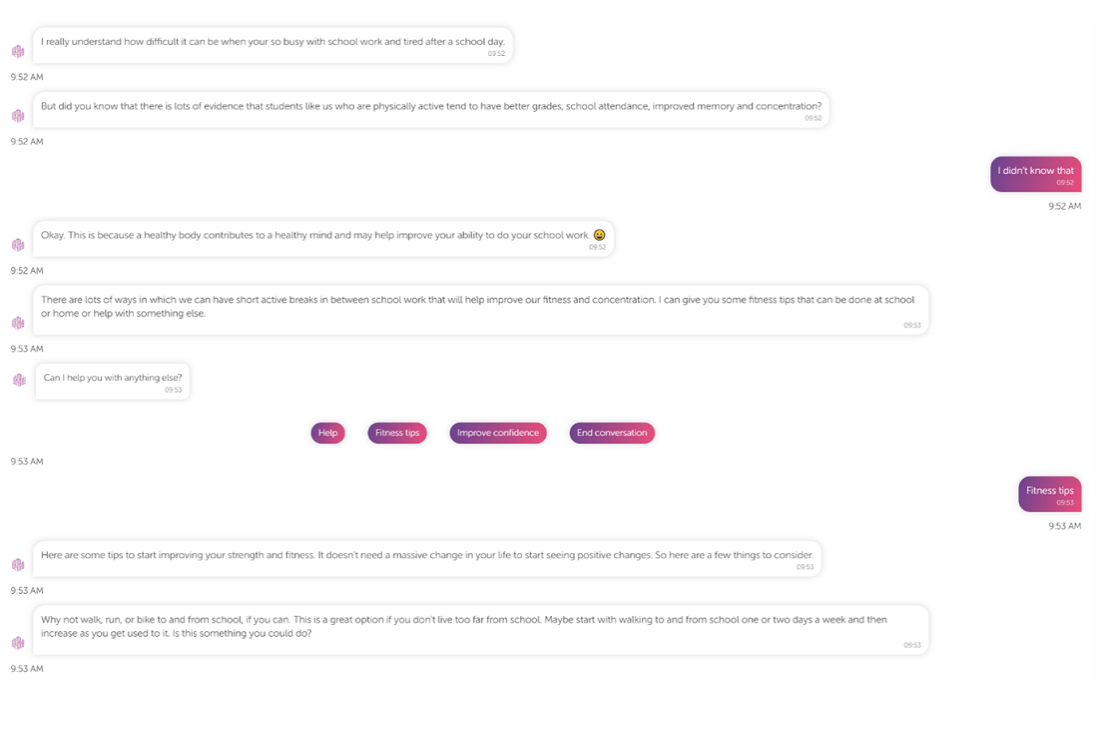
An example of an interaction between a student and the conversational agent.

Figure 1 provides an example of an interaction between a student and the CA.

To assess the accuracy of the model, the researchers used MindBehind, a software program that enabled the research team to input statements pertaining to each barrier and evaluate and refine the utterances used during the NLU model training. The performance of the CA was evaluated using an *F*_1_-score, yielding a score of 80% [48]. MindBehind was then used to create a knowledge model and use its conversational engine, leveraging the NLU model and logic functions to route conversations based on the input of barriers and corresponding solutions. Following this, the researchers visually represented the conversational dialogue of the CA (ie, barriers and solutions) on a canvas and tested its accuracy to ensure its readiness to be deployed for testing.

#### Phase 3: Evaluating Mechanisms and Outcomes

During the final phase of the study, the CA was distributed to the students for testing via a test link. The students interacted with the CA through text messaging. The platform hosting the CA enabled the students to engage with it through various internet-enabled devices such as laptops, tablets, and smartphones. In addition, the students had the option to select appropriate responses from a predefined list of choices, which included options such as requesting help, ending the conversation, or seeking more support. This setup provided a user-friendly and versatile interface, accommodating different communication preferences and ensuring a seamless interaction experience for the participants. Initially, the adolescents were asked to engage in a conversation with the CA as users and to identify any barriers they faced that led them to seek assistance from the CA. Subsequently, they were requested to test the CA by inputting various barriers and assessing how the conversation was routed. Following this, participants were invited by each organization using convenience sampling to evaluate the CA from Wickersley School and Sports College (7 participants) and a community enterprise called Zest (31 participants), totaling 46 adolescents.

To evaluate the CA’s acceptability, usability, and perceived outcomes of using the modules, the users were invited to participate in a web-based survey using Key Survey, a specialized web-based survey platform. The survey also sought to gauge their perception of the CA’s effectiveness and user experience, encompassing 7 recommended themes for evaluating CAs, as outlined in a previous study [29]. Users were specifically encouraged to share both positive and negative aspects of their experience, offer suggestions for improvement, and report any observed malfunctions during their engagement. In addition, the survey included an adapted version of the AttrakDiff questionnaire, tailored specifically for young individuals, to measure usability [49].

### Analysis

During phases 1 and 2, an analysis was conducted on both quantitative and qualitative data gathered from the participants. For the quantitative analysis, basic frequencies were used to examine data related to students’ age, sex, and levels of PA in phase 1 and to evaluate the user experience and efficacy of the CA in phase 2. This analysis provided an overview of the demographic characteristics and the distribution of PA levels among the participants, as well as an objective assessment of their experience and the outcomes of using the CA.

The qualitative data were subjected to thematic analysis [36] to identify and analyze recurring themes and patterns across several key aspects. These aspects included the persona and key characteristics that the CA should possess in phase 1, as well as user feedback on how the CA could be improved in phase 2.

### Ethical Considerations

This study adheres to the established human subject research ethics guidelines and was granted institutional ethics approval (ER37229351) by the Sheffield Hallam University Research Ethics Committee.

Informed consent was obtained from participants’ legal guardians following a comprehensive explanation of the study’s purpose, procedures, risks, benefits, and the voluntary nature of their participation. The participants were explicitly informed that they had the right to withdraw from the study at any time without repercussions.

The privacy and confidentiality of all human subjects were rigorously safeguarded throughout the study. All the data collected were either anonymized or deidentified to protect the identities of the participants. The data were stored securely, and the data used in this study were not linked to any external databases or sources that could compromise the privacy of the participants. The participants were not offered an incentive to participate in the study.

## Results

### Co-Design

The following section presents the results of the co-design with adolescents and the evaluation of the performance of the CA and its efficacy.

#### User Background

The co-design of the CA involved 9 students aged between 11 and 13 years, in school years 7 to 9. The sex distribution among the students was 56% (5/9) female and 44% (4/9) male. In terms of ethnic background, 6 (67%) of the 9 students identified as White, 2 (22%) identified as Asian or Asian British, and 1 (11%) identified as Black.

All 9 students met the inclusion criteria and expressed their interest in exploring the use of a CA to support themselves and their peers to help overcome barriers to PA. Initially, the students were provided with a list of 52 barriers to PA and were asked to identify the specific barriers that they experienced. They highlighted 37 barriers, which accounted for 66% of the total barriers listed in the previous study [17].

Among the barriers identified, the most commonly highlighted included a “lack of confidence” (mentioned by 8 students), “fear of failure” (mentioned by 7 students), and a “lack of motivation” (mentioned by 5 students). When the adolescents were asked to elaborate on the barriers in their own words, they provided more specific insights, particularly concerning psychological barriers. For instance, 2 adolescents reported experiencing “anxiety” both before and during PA, especially at competitive events. Other students mentioned feeling self-conscious when being judged by others, lacking confidence in their abilities, feeling unmotivated because of excessive phone use, and harboring fears of getting injured. Given that these barriers were already included in the previously identified 52 barriers, they were all integrated into the programming of the NLU model for the CA.

The students were asked via the workbook to identify and express their emotions related to their experiences with PA. This information played a vital role in shaping the design of the modules and the conversational language used to establish relational capacity with the users. On average, the students reported experiencing 6 positive emotions and 9 negative emotions in relation to PA. A total of 21 distinct positive emotions were identified, with the most frequently mentioned being pride (mentioned by 5 students), optimism, and confidence (mentioned by 4 students each). In contrast, negative emotions were highlighted significantly more often (84 times), with the most frequently mentioned being humiliation, stress, insecurity (mentioned by 5 students each), and embarrassment (mentioned by 4 students).

Most students expressed an ardent desire to engage in more PA and recognized the numerous benefits associated with it. However, they also identified negative thoughts and emotions as significant barriers to their participation in PA. Notably, students mentioned using PA to enhance self-esteem, productivity, confidence, and feelings of empowerment and strength, in addition to its health benefits. This apparent paradox of wanting to be more active in improving confidence while simultaneously lacking the confidence to do so presents a unique global challenge that the proposed CA aims to address.

The students who participated in the study acknowledged the importance of receiving support to overcome the barriers they faced in engaging in PA. Despite having diverse experiences with CAs in the past, they demonstrated an interest in exploring the potential of using a CA for this purpose. It was evident that the students lacked access to adequate support systems to address their barriers, which highlights the innovative nature of the proposed approach. Despite this, the students displayed receptiveness and optimism toward using the CA to overcome these barriers. They also demonstrated a willingness to share personal information with the CA. However, it is worth noting that some students expressed concerns regarding privacy and data protection; as such, these concerns should be carefully considered and addressed during the future development and implementation of the CA to ensure user trust and data security.

#### Relational and Persuasive Capacity

During the co-design process, the students were actively involved in expressing their preferences for the persona and characteristics of the CA. All co-design group members unanimously agreed that the CA should fall within the age range of 11 to 18 years, with the majority believing that a teenage persona would be most relevant and engaging for the target audience. When it came to gender, of the 9 students, 4 (44%) expressed a preference for a gender-neutral CA, whereas only 1 (11%) person advocated for a gender-specific CA. The remaining students (3/9, 33%) suggested that the CA’s gender should be customizable, allowing users to select their preferred option. In terms of appearance, most students felt that if the CA had an avatar, it should be customizable, with suggestions ranging from human-like features to animal-like or robot-like attributes.

Regarding the tone of the CA, approximately two-thirds of the group agreed that it should have a human-like and engaging tone, regardless of whether it was in the text or voice form. Of the 9 students, 2 (22%) students even proposed that the CA could mimic the tone of a coach or celebrity. Conversely, 1 (11%) person expressed a preference for the CA to have a robotic voice. However, 3 (33%) individuals indicated a preference for customizable voices, although it remained unclear whether these voices should be exclusively human-like.

During the design phase, the concept of customization and personalization emerged as a prominent theme, reflecting the students’ desire to have control over various aspects of the CA to meet their individual needs. This preference for customization poses several design challenges, as it necessitates the creation of multiple personas and the development of appropriate messages for each persona. However, it is a crucial consideration for the future development of CAs, as it allows for genuine personalization and enhances the overall user experience.

As part of the design process, students were also invited to suggest names for the CA. The name suggestions varied, highlighting different preferences and creative ideas. Some students proposed generic brand names with a playful twist related to PA, such as “Sporta” or “Phylo.” Others suggested human names such as Robert or Patricia, whereas some students preferred names that explicitly incorporated the terms “CA” or “robot,” such as “Charlie the CA” or “Sporty Bot.”

For the prototype, only 1 persona was created, and it was named “Phyllis.” This name remained unchanged throughout the final testing phase, as some students expressed a liking for the name, whereas others did not find the other suggested names suitable. The decision to choose Phyllis as the chosen name for the CA reflects the consideration of participant preferences and the desire for consistency during the testing phase.

**Figure 2:**
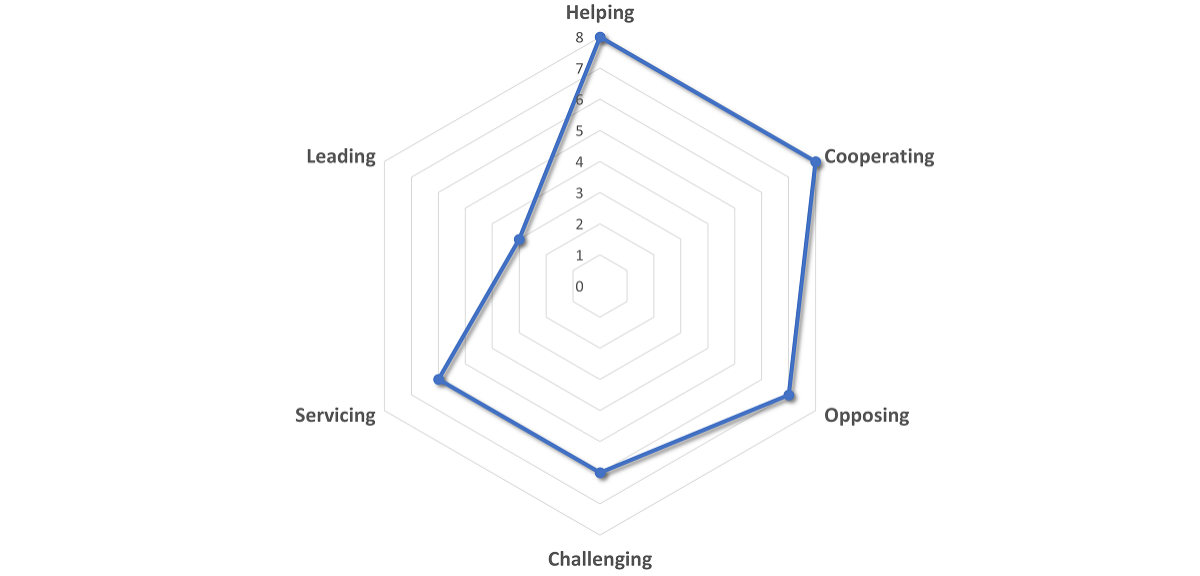
Leary’s Interpersonal Complex results.

During the co-design process, the students were asked to indicate their preferred personality characteristics for the CA using Leary’s Interpersonal Complex (Figure 2), which measures personality along 2 dimensions: dominance (horizontal) and agreeableness (vertical). As shown in Figure 2, the results revealed that students sought a balanced combination of dominance and agreeableness in the CA’s personality. They desired a friendly CA that exhibited both submissive and dominant behaviors and even opposing viewpoints at certain points in the conversation, potentially to challenge the user’s beliefs. These findings align with the broader characteristics that the students identified during the co-design process, emphasizing their need for personalization.

In total, the students selected 30 distinct characteristics that they believed the CA should possess, underscoring their desire for a personalized and tailored experience. Among the co-design participants, only 3 characteristics were mentioned more than once: understanding (mentioned 2 times), intelligence (mentioned 3 times), and kindness (mentioned 3 times). These repeated characteristics indicate the significance of empathy, intelligence, and kindness in the preferred personality traits of the CA.

Figure 2 illustrates students’ preferred personality characteristics using Leary’s Interpersonal Complex. The graph measures personality along 2 dimensions: dominance (horizontal) and agreeableness (vertical).

#### Social Dialogue and Self-Disclosure

Students were asked to provide dialogue examples that represented the personas they had chosen for the CA. All personas were of peer age for the students. The dialogue examples covered various scenarios, including greetings, providing assistance, encouraging positive actions, offering reassurance, giving praise, acknowledging mistakes, and saying goodbye.

The welcome messages displayed personalization, often addressing users by their first names and minimizing small talk. The style of the messages was similar across the personas. The “help” messages were typically direct and straightforward, presenting suggestions (eg, “I have a suggestion”). The “take positive action” messages were motivational, encouraging users to take specific actions (eg, “You can do it! How about you try this?”) Reassuring messages often began with an “okay” to acknowledge the user’s concerns before offering support. “Praise” messages consistently started with “well done” and then reiterated the user’s accomplishments. The “goodbye messages” usually included a supportive or hopeful closing message to conclude the conversation.

This approach to social dialogue and self-disclosure, characterized by personalization, motivational language, acknowledgment of concerns, and positive reinforcement, was preferred by the co-design participants. Their preferences were considered and integrated into the dialogue design of the CA persona.

#### Content

As part of the co-design process, students expressed their preferences regarding the content they wanted to be delivered through the CA, focusing on the barriers to PA they had previously identified. Most students preferred content that would help boost their confidence, overcome insecurities, address problems, and manage negative emotions related to PA. A student (1/9, 11%) suggested that the CA should provide advice on supporting others, whereas another (1/9, 11%) preferred a more proactive approach in which the CA would listen, understand their problems, and provide relevant answers.

Different preferences emerged regarding the approach the CA should adopt to engaging with adolescents and delivering content. A student (1/9, 11%) preferred a guiding approach, allowing the adolescents to find their own solutions while also offering assistance if they struggled with that approach. Practical solutions were also highlighted, such as suggestions for various sports activities, strategies to stay physically active, ways to improve performance in sports, and tips for eating healthier. Furthermore, the students recommended including motivating quotes and information on accessing support through other organizations or websites.

#### Media

The students were provided with diverse options to choose from regarding the type of media they would like to use during the CA conversation. According to their preferences, the most favored options were pictures (7/9, 78%) and videos (7/9, 78%), followed by audio (6/9, 67%) and links to websites. Conversely, the least popular options were links to apps, social media (2/9, 22%) and documents (1/9, 11%). The CA being piloted incorporates pictures, animated pictures (GIFs), and links to websites and apps, whereas videos were not included owing to the lack of appropriate content for adolescents, corresponding to the barriers identified. In the future, filming bespoke videos would be necessary to provide engaging content to adolescents via the CA.

#### Dialogue Infrastructure

Owing to the students’ age and unwillingness to disturb their schooling, the researchers were unable to spend extended periods with them and had to rely on the students’ prior experience with CAs. In the future, it is recommended that more time be spent with the students to fully understand all the key features of CA design and improve the co-design process.

#### Implementation of Co-Design Phase Recommendations

In this section, we summarize the implementation of the findings from the co-design process in the development of the prototype CA.

Textbox 1 presents the summarized findings from the co-design research with 8 adolescents to develop the prototype chatbot. It details the findings of user requirements and context, the content of the chatbot, and the chatbot’s persona.

**Summary of co-design findings.**

**Conversational agent (CA)**

- Name: Phyllis
- Audience: Students aged 11-16
- Channel: A school website or social media

**Use case**

- Service and expertise
  Identify barrier to physical activity (PA) from user input.
  Provide solution to barriers including recommending activities they could participate in.
  Prototype provides 2 modules on motivation and confidence.
  Future version will have solutions to all 52 barriers as well as monitoring of PA.
- Purpose
  Support for students in schools to overcome barriers to PA and to understand more about PA and gain information and guidance of how to be physically active.
- Media
  CA mainly conversational but provides links to other website apps and resources.
  Future iterations will include voice and video media.

**User requirements and context**

- User persona
  Students aged 11-16 that want to be more active or support others to be active
- Motivations
  Want to be more physically active but aware that there are barriers which prevent them from participating in PA and they want to overcome these
- Anxieties
  That the CA can understand them
  That the CA can help them
  Data protection

**Content**

- Behavioural modules
  Improving confidence (5 min)
  Improving fitness (4 min): General fitness
    Balance with educational constraints
    Links to Sport England content
    Fitness tracking
- General content
  Why it is important to be physically active
  How long should adolescents be active for
  Respond to input around 52 barriers to PA. Currently acknowledges and asks to confirm barrier before saying content is not yet available.

**Bot persona**

- Gender and age
  Age: 16
  Gender: Neutral
  Future designs may include a choice of personas
- Backstory
  An understanding intelligent and kind young person with knowledge and experience of PA
- Role and style
  Helpful peer who is supportive to other students of a similar age
- Personality
  Humanoid
  Helpful
  Challenging
  Understanding
  Intelligent
  Kind
- Can do
  Have a conversation and identify barriers to PA
  Suggest solutions to barrier and ways to be more active
  Provide general information about PA
- Can’t do
  Have a wider conversation than the stated use case
  Advise on how to help others
  Monitor PA

**Standard vocabulary**

- Typical things to say
  Here are some ideas.
  I have a suggestion.
- Introductions
  Hi, my name is Phyllis how are you?
  Hey, my name is Phyllis. 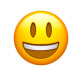

  See you soon.
- Acknowledgement
  Sure, I will do that.
  Okay, I have a suggestion.
- Confirmations
  Here you go ‘name’.
  You can do it! How about you try this.
- Apologies
  I am sorry I cannot help you with that.
  Okay, I didn’t understand that. Can you try and tell me what stops you from being more physically active?

Following the co-design process, the CA was constructed based on the gathered findings and was internally evaluated with the research team. Subsequently, the CA was prepared for deployment to be tested with adolescents.

### Evaluation

This section presents the findings of evaluating the prototype CA (referred to as “Phyllis”) with 46 students.

#### Use Patterns

During the evaluation phase, 62 conversations were conducted with the CA. Among these conversations, 15 students concluded one conversation and then initiated a new conversation with Phyllis. Overall, the users sent 1512 messages to Phyllis, resulting in an average of 33 messages per person. Phyllis responded by sending 2806 messages to the users, averaging 61 messages per conversation. Only a small portion (n=77, 5.09%) of the messages sent per person could not be successfully matched with a corresponding intent. This was because the students sent unintelligible messages or misspelled words while discussing barriers to PA participation. The most frequently matched intents included “lack of confidence,” “lack of time,” and “lack of motivation,” which aligns with the barriers identified during the co-design focus groups.

#### User Experiences

**Figure 3:**
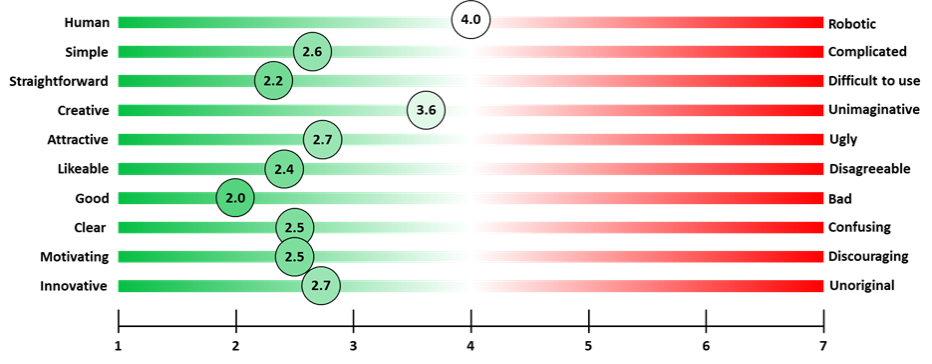
User experiences and relational capacity.

The users generally had positive experiences with the CA, rating its conversational capacity as good, clear, simple, and straightforward to use on a comparative scale (Figure 3). Qualitative feedback indicated that the users found the CA clear and easy to use, with good options for obtaining information and interesting websites to access from the CA.

Concerning acceptability, a sizable portion of the students (34/46, 73%) expressed their definite intention to use a fully functional CA in the future. The remaining students stated that they might consider using it in the future. When asked to rate Phyllis, 65% (30/46) of respondents considered it “good,” whereas 20% (9/46) rated it as “very good.” The remaining 15% (7/46) rated Phyllis as “average.” The CA received a positive net promoter score of 13, indicating a favorable likelihood of recommendation. This suggests that not only would most students use Phyllis themselves but also they would recommend it to their friends.

On the basis of user feedback, it was suggested that Phyllis should reduce the amount of text in each message to enhance readability and recommended slowing down the speed of message delivery to assist with user recognition and comprehension. Furthermore, 2 users specifically requested that the CA offer direct answers to various questions about fitness and exercise without necessitating the initial mention of barriers. They also suggested incorporating more videos, diagrams, and real-life scenarios depicting PA. As anticipated, users expressed an interest in customizing the CA further by having the option to choose a different avatar, name, and personality.

Figure 3 provides a visual representation of students’ user experience with Phyllis and evaluates the relational capacity of the CA using a 7-point Likert Scale across 10 themes. Scores range from 1 (indicating high satisfaction) to 7 (representing the lowest satisfaction).

#### Relational Capacity

The students assessed the CA’s relational capacity and reported finding the bot to be “likable” and “motivating.” However, the scores for the persona of the CA being either “human” or “robotic” and “creative or unimaginative” were neutral, warranting further investigation. Qualitative feedback revealed that certain students appreciated the bot’s personality; humor; and use of emojis, icons, and GIFs. They found Phyllis to be helpful, informative, and reassuring, particularly highlighting Phyllis’s use of open-ended questions. A participant (1/46, 2%) even expressed gratitude for Phyllis being a peer, stating, “students like us” during the conversation. However, some students felt that Phyllis could offer more support and advice, particularly within the fitness module. Rather than receiving a list of websites, they expressed a preference for conversational and diverse guidance on engaging in PA.

#### Persuasive Capacity

A total of 2 modules, focusing on the behaviors of “confidence in user ability to engage in PA” and “motivation to be physically active,” were evaluated. Users were asked to rate their agreement level with 3 statements, both before (pre) and after (post) the use of Phyllis. A total of 80% (37/46) of students reported being “confident in their ability to engage in PA or play sports,” indicating a 50% increase from the initial preresponse rate of 53%. The findings for motivation were even more notable, with 73% (34/46) of users reporting an enhanced motivation to engage in PA following the intervention. This represents a significant 120% increase in agreement with the statement from 33% (15/46) pre- to 73% (34/46) postintervention. Furthermore, students were asked about their ability to overcome challenges related to PA or sports. Over half of the respondents (24/46, 52%) agreed that they could overcome these challenges, which increased to 67% (31/46) after engaging with Phyllis. However, it is worth noting that this outcome may have been less substantial owing to the current limitations of the CA, as it is unable to provide solutions or modules for all 52 identified barriers. Overall, 67% (31/46) of respondents felt more motivated to participate in PA or sports since using Phyllis, and an overwhelming 93% (43/46) of them expressed a desire to be more active throughout the day. In addition, 73% (34/46) of respondents indicated that Phyllis helped them contemplate ways to increase their PA levels during the day.

Overall, there was no significant difference reported between the adolescents who were part of the co-design and those who were not. This was the same for users when comparing users from Wickersley School and Sports College and Zest.

## Discussion

### Principal Findings

The study involved co-designing and evaluating a pilot CA named Phyllis to test its proof of concept. The primary objective was to support adolescents in overcoming barriers to PA [15-17], to realize the associated benefits [1,2,4,50], and to provide an alternative digital solution to combat global inactivity [6]. Furthermore, it aims to promote the development of healthy behaviors during adolescence [7,8,51], thereby fostering continued participation in adulthood. The hypothesis guiding the study was that a CA could assist adolescents and that they would perceive Phyllis as a valuable tool for boosting their confidence and motivation to engage in PA. The study’s objectives were to co-design the CA in collaboration with adolescents, demonstrating the CA’s capacity to understand user input related to the identified barriers. It also aimed to evaluate the CA’s usability and acceptability among adolescents and assess the perceived effectiveness of the solutions offered by the CA to address these barriers. Overall, adolescents reported high acceptability and positive user experiences when using Phyllis, whereas the modules and personas designed to increase motivation and confidence achieved positive outcomes. This evidence positions Phyllis as a promising digital tool to alleviate global inactivity and aligns with the goals of the World Health Organization’s Global Action Plan on PA [18].

The research builds upon prior studies of PA interventions [24,25,27,28,52,53] and adolescent barriers to PA [15,16,51] and is informed by a behavior change model and theoretical framework [29]. This approach is unique as it draws upon relevant research, engages in co-design with adolescents and applies relevant theories [30,35,38,54] to design a CA targeted at adolescents. It is a novel approach in this domain targeted at this population, as indicated by prior research [27,28], which has identified a lack of CAs tailored specifically for adolescents. This innovation in design, using the COM-B model and TDF for intervention development, promises to pave the way for more impactful interventions in the future.

The co-design process engaged 8 adolescents, which represented the broader population’s sex distribution and a higher representation of “Asian” and “Black” ethnic groups who are more likely to be inactive [17]. The process yielded significant findings that support the rationale for the study. First, it highlighted the inadequate access that adolescents have to support systems for addressing barriers to PA and provided further evidence of the array of barriers faced by adolescents [15-17]. Co-design participants were able to identify 37 barriers to engaging in PA, reporting an average of 9 barriers per person. This accounts for approximately 66% of the barriers that the CA will aim to support, based on a study that served as the foundation for the current research [17]. These findings highlight the significance of developing multicomponent interventions that can effectively address multiple barriers, as advocated in the existing literature [12]. Notably, a lack of confidence and motivation emerged as significant barriers to this cohort, which is consistent with previous studies [15,16,51]. Moreover, adolescents reported experiencing 4 times as many negative feelings as positive feelings when describing the emotional impact of PA, highlighting potential reasons for limited engagement in PA. An unintended outcome of the study was the revelation of a paradox, as some adolescents reported that PA may exacerbate psychological effects such as anxiety, while perceiving it as a means of improving these conditions. Subsequent iterations of Phyllis will consider these limiting beliefs, aiming to help individuals become more self-aware of such conflicting thoughts.

For the CA to effectively address barriers to PA, it must be perceived as an acceptable tool for support by adolescents. The co-design participants expressed high levels of acceptability toward the CA and demonstrated a willingness to share personal information with it. However, some students voiced concerns regarding privacy and data protection, aligning with the findings of other studies [27]. Previous studies have highlighted the significance of personalization [23], and this study further underscores adolescents’ specific requirements. Although a consensus favored a gender-neutral teenage persona with a human-like tone, the ideal scenario would involve fully customizable features such as persona, avatar, and tone of voice. The adolescents’ diverse preferences were also reflected in their choice of the CA’s personality and characteristics, representing a range of personalities. Although challenging for designers, this evidence is valuable for guiding the future design of CAs and for advancing research in this domain. Overall, the feedback emphasized the importance of empathy, intelligence, and kindness in the CA’s approach, characterized by motivational language, acknowledgment of concerns, and positive reinforcement through both submissive and dominant behaviors at specific points of interaction. For example, some adolescents appear willing and would even encourage the CA to challenge their beliefs and offer alternative views and solutions. Similarly, there was variation in the preferred approach to delivering content using different media; yet, it was clear that adolescents desired more complex interactions with content delivered and explained conversationally rather than being signposted to external websites or apps This perception strengthens the case for CAs to be used for this purpose, with interventions delivered conversationally. All this knowledge provides much-needed insight into the design of effective CAs targeted at this population.

The second objective was to build a CA that, although informed by the co-design, had a specific purpose of recognizing input from adolescents around the 52 barriers [17] and providing a relevant solution to overcome each barrier. This is a novel approach that has not been applied in the design of previous CAs. In terms of recognition, an *F*_1_-score was used to assess the performance of the model, which scored 80%. All relevant and negligible utterances were matched accurately during the pilot, and adolescents who typed utterances related to “a lack of confidence” or “fitness” were accurately matched to the solution. Further testing will be required when all solutions are developed, including training data, to help improve recognition further. Importantly, the model can recognize a range of input from adolescents, thereby improving the potential viability of this approach to support adolescents.

Once the prototype CA was designed and developed, the objective was to validate it as a proof of concept by assessing its acceptability, usability, and effectiveness in supporting adolescents to overcome barriers to PA. Most adolescents expressed positive acceptability toward the CA, with 73% (34/46) of students indicating they would “definitely use” a fully operational Phyllis and 85% (39/46) rating the CA as either “good” or “very good.” In addition, Phyllis received a “good” net promoter score, indicating that adolescents would also recommend it to their friends [55]. Quantitative data analysis revealed positive scores in terms of user experience, whereas qualitative feedback highlighted students’ appreciation for the CA’s personality; humor; and the use of emojis, icons, and GIFs.

The evaluation also provided valuable feedback to enhance Phyllis in the short term (eg, delivering fitness activities conversationally) and long term (eg, enabling deeper personalization and reducing message text). Students found Phyllis helpful, informative, likable, and motivating and appreciated its ability to ask open-ended questions, demonstrating the importance of unconstrained conversation [28] and the ability to provide reassurance. These results indicate that adolescents had a positive user experience and would be willing to use a CA such as Phyllis in the future, further strengthening the evidence for these tools to be used to support adolescents. Such features developed from the co-design process will help alleviate concerns expressed in the literature around usability challenges [27] faced by users and further reinforce the importance of integrating the principles of user-centered design [30] and human-computer interaction [31] with the aim of establishing relational and persuasive capacity [28] with users. These findings could also be used to inform the development of other CAs in this and other domains, serving as useful insights for designers and developers of digital health interventions.

The primary goal of the CA is to augment PA behaviors, and the prototype included 2 modules developed for this purpose. Following an interaction with the CA, 80% (37/46) of the students reported improved confidence in their ability to participate in PA or sports. This represents a 50% increase compared with the initial response. In addition, there was a significant 120% increase in the proportion of students who agreed with the statement expressing their motivation to engage in PA after engaging with the CA. This serves as a positive indicator of the CA’s efficacy. Most students (34/46, 73%) also indicated that the CA would assist them in exploring ways to incorporate more PA into their daily routines. These findings demonstrate the potential of the CA as an effective tool in helping students overcome barriers to PA, further validating the CA’s potential. However, further research is necessary to determine if these improvements translate into increased levels of PA and to identify an appropriate measurement method so that PA can be monitored via the CA.

It is important to consider the implications of these findings in the context of recent developments in LLMs and generative AI, as they have the potential to enhance the effectiveness of the CA as they can comprehend and generate natural language with heightened complexity and accuracy [33,34]. These advancements can also expedite the development process, elevate the quality of interaction, and expand their knowledge base by providing a more comprehensive conversational experience that meets the specific needs of users. In health-related fields, it is imperative that CAs provide accurate, trustworthy, and evidence-based information and address concerns related to bias, misinformation, privacy, and security that may arise from the use of LLMs.

The approach advocated in this study has the potential to bridge the gap between sophisticated LLMs and trusted, evidence-based content. To illustrate this further, interventions should continue to be co-designed and expert led, grounded in evidence-based content. This content can then serve as the foundation for the development of domain-specific models, which in turn are used to provide data to inform intervention delivery. The critical aspect of intervention delivery depends on the use of prompt engineering or the application of a cognitive system that guides the model on how and when to deliver an intervention. The process of delivering interventions may necessitate extremely precise prompting to ensure the accuracy of behavior change tools, intervention content, language, and persona. This precision may require the use of an LLM working in combination with a traditional intent-based approach to allow for predefined responses to guarantee the accuracy and quality of the expert-designed intervention content. Adhering to these principles can effectively mitigate concerns and establish an audit trail for the content provided, ultimately promoting greater transparency. This transparent and comprehensive audit trail serves not only to foster trust but also to strengthen accountability and facilitate the identification of potential biases or inaccuracies.

The limitations of the study findings lie in their reliance on a single interaction with the CA, which does not provide insight into long-term adherence or sustained behavior change resulting from CA use. Nonetheless, the results show promise in terms of children’s willingness to engage and to self-report positive attitude changes after a brief period of interacting with the CA. The evidence also demonstrates why this population perceives CAs as a potentially valuable solution. Further research is necessary to address each of the 52 identified barriers, identify the commonalities among them, enhance the algorithm, and ensure greater efficacy.

Overall, the results demonstrate that Phyllis has the potential to be a cost-effective, resource-efficient solution that organizations can offer to support adolescents and address the multifaceted barriers to PA [15-17]. With further development, the tool could serve as a self-help resource for students, with teachers administering it to inactive students to enhance their levels of PA. Positive evidence of digital interventions being used in both formal and informal settings exists to support their potential use in this setting [22]. As this research highlights, schools lack the time, resources, and capacity to adequately support adolescents [14], making the CA an effective tool to augment existing support. Data from CA interactions could also be shared with schools to identify more serious barriers such as mental and physical health issues or to highlight prevalent barriers within the student population, thereby improving support within the CA and informing policy or intervention approaches within schools. Moreover, the data could offer greater insight into adolescent needs and barriers, informing policy in this field.

The implementation of digital technology in schools, which ensures transparency, safety, and trust, can be achieved with minimal demands on school staff and teachers while also being cost-effective. The findings reinforce the importance of evidence-based self-help tools, which can be accessed by adolescents in schools at a low cost. Moreover, these tools can be supervised by trusted adults, ensuring personalized support for adolescents without overwhelming the capacity of teachers or the school. The CA and its design also have the potential for broader deployment within health care systems as part of the PA referral pathway to enhance adherence to programs or social prescribing services that aim to enhance PA levels among adolescents with long-term conditions or hypokinetic diseases. The design could also be enhanced to provide multimodal support by using robotics and be adapted and used within other populations, such as with older adults.

### Conclusions

The study aimed to co-design, develop, and evaluate a prototype CA as a proof of concept to assist adolescents in overcoming barriers to PA. It presents one of the first theory-driven approaches to designing a CA. Drawing from prior studies, theoretical approaches, and insights into adolescent barriers, the research focused on achieving relational and persuasive capacity with adolescents using the CA. Highlighting inadequate access to support systems, 37 barriers were identified, with 66% aligning with previous research. The emphasis was placed on the acceptability and personalization of the CA to address privacy and data protection concerns. The results demonstrated high acceptability and positive user experiences, highlighting the potential of the CA. The modules designed for the CA showed promising outcomes, fostering increased confidence and motivation for PA. Phyllis also performed well, recognizing a range of different barriers to PA. However, further research is needed to develop other modules to overcome other barriers and explore long-term adherence and the effectiveness of interventions for sustaining behavior change. Adolescents also expect to have a personalized experience and be able to personalize all aspects of the chatbot. Phyllis holds potential as a cost-effective solution for schools to support adolescents and tackle barriers to PA. The integration of LLMs can significantly enhance the CA’s capabilities, facilitating sophisticated conversations and automated content generation with this study providing knowledge of how they can be designed to incorporate evidence-based approaches to ensure trust and transparency. The CA’s potential extends to health care systems, social prescribing services, and multimodal support, including robotics. This study provides valuable insights for designing and developing digital health interventions in other domains as well as contributing to the improvement of PA levels among adolescents and addressing global inactivity concerns.

## Abbreviations

AI: artificial intelligence
CA: conversational agent
COM-B: capability, opportunity, motivation, and behavior
LLM: large language model
NLU: natural language understanding
PA: physical activity
TDF: Theoretical Domains Framework

## Data Availability

The data sets used and, or analyzed during this study are available from the corresponding author upon reasonable request.
